# miR-152-3p aggravates vascular endothelial cell dysfunction by targeting DEAD-box helicase 6 (DDX6) under hypoxia

**DOI:** 10.1080/21655979.2021.1959864

**Published:** 2021-08-10

**Authors:** Zhongyan Zhao, Chanji Wu, Xiangying He, Eryi Zhao, Shijun Hu, Yeguang Han, Ting Wang, Yanquan Chen, Tao Liu, Shixiong Huang

**Affiliations:** a Department of Neurology, Hainan General Hospital (Hainan Affiliated Hospital of Hainan Medical University), Haikou, Hainan, China; b Department of Central Laboratory, Hainan General Hospital (Hainan Affiliated Hospital of Hainan Medical University), Haikou, Hainan, China

**Keywords:** Mir-152-3p, ddx6, hypoxia, angiogenesis, endothelial cell permeability

## Abstract

Stroke is a main cause of disability and death worldwide, and ischemic stroke accounts for most stroke cases. Recently, microRNAs (miRNAs) have been verified to play critical roles in the development of stroke. Herein, we explored effects of miR-152-3p on vascular endothelial cell functions under hypoxia. Human umbilical vein endothelial cells (HUVECs) were treated with hypoxia to mimic cell injury *in vitro*. Reverse transcription quantitative polymerase chain reaction revealed that miR-152-3p exhibited high expression in HUVECs treated with hypoxia. The inhibition of miR-152-3p reversed hypoxia-induced decrease in cell viability and the increase in angiogenesis, according to the results of cell counting kit-8 assays and tube formation assays. miR-152-3p inhibition reversed the increase in endothelial cell permeability mediated by hypoxia, as shown by endothelial cell permeability *in vitro* assays. In addition, the increase in protein levels of angiogenetic markers and the decrease in levels of tight junction proteins induced by hypoxia were reversed by miR-152-3p inhibition. Mechanistically, miR-152-3p directly targets 3ʹ-untranslated region of DEAD-box helicase 6 (DDX6), which was confirmed by luciferase reporter assays. DDX6 is lowly expressed in HUVECs under hypoxic condition, and mRNA expression and protein level of DDX6 were upregulated in HUVECs due to miR-152-3p inhibition. Rescue assays showed that DDX6 knockdown reversed effects of miR-152-3p on cell viability, angiogenesis and endothelial permeability. The results demonstrated that miR-152-3p aggravates vascular endothelial cell dysfunction by targeting DDX6 under hypoxia.

## Introduction

Stroke is the second cause of death and the primary cause of disability in adults worldwide [1]. Stroke is characterized by the failure of blood flow to complete normal cerebral circulation and thus results in oxidative stress and inflammatory injury to brain tissues [2]. Stroke can be classified into ischemic stroke and hemorrhagic stroke [3]. Ischemic stroke, as the most common type of stroke, is responsible for 80% of all stroke cases [4], which is resulted from the lack of blood flow that contains oxygen and nutrients [5].

The decrease in blood supply after stroke can activate angiogenesis, which is a compensation for the lack of oxygen and is necessary for recovering neurovascular injuries [6]. Previous studies have verified that angiogenesis exerts critical functions in improving neurological functional recovery after stroke [7,8].

Endothelial function is essential to maintain normal brain function, and the endothelium of brain vasculature serves as blood-brain barrier (BBB). The failure of BBB can be characterized by levels of tight junction proteins such as zonula occludens-1 (ZO-1) and occludin are downregulated due to ischemia [9–11]. Therefore, the decrease in ZO-1 and occludin protein levels can be deemed as a biomarker of endothelial dysfunction [12].

Mature microRNAs (miRNAs) are transcripts containing approximately 18–22 nucleotides in length [13]. miRNAs can regulate the expression of downstream messenger RNAs (mRNAs) post-transcriptionally, which are involved in the development of individuals or diseases [13,14]. Specifically, miRNAs bind with 3ʹ-untranslated regions (3ʹ-UTR) of mRNAs to accelerate the degradation of mRNAs [15]. Emerging evidence has revealed that miRNAs are significant regulators of endothelial cell functions [16,17]. For example, miR-124-3p overexpression was discovered to suppress the proliferation and migration of HUVECs as well as angiogenesis *in vitro* [18]. miR-182 promotes BBB failure by exacerbating endothelial cell apoptosis via the mTOR/forkhead box O1 (FOXO1) pathway during cerebral ischemia [19]. miR-874-3p overexpression inhibits C-X-C motif chemokine ligand 12 (CXCL12) expression to promote angiogenesis in HUVECs [20]. Herein, we intended to explore effects of miR-152-3p on functions of vascular endothelial cells under hypoxia. According to previous investigation, miR-152-3p overexpression promotes the proliferation of mammary epithelial cells [21]. In addition, miR-152-3p has been verified to show low expression in the brain tissues of mice treated with ischemic/reperfusion [22] and in patients with acute ischemic stroke [23].

We herein aimed to explore the effects of miR-152-3p on vascular endothelial cell viability and permeability as well as angiogenesis *in vitro*. In addition, the miRNA-target network in HUVECs was also under investigation. We hypothesized that miR-152-3p may affect vascular endothelial cell dysfunction by targeting certain downstream mRNA. The study may provide novel insight into the role of miRNAs in vascular endothelial cells.

## Materials and methods

### Bioinformatics analysis

The mRNAs containing binding site with miR-152-3p were predicted using the starBase (http://starbase.sysu.edu.cn/) [24]. Six mRNAs (DDX6, PNPLA6, ATP2A2, QKI, SLC25A44, GADD45A) were identified under the condition of Cross Linking and Immunoprecipitation-sequence (CLIP) Data: strict stringency (≥5); Degradome Data: high stringency (≥3); Pan-Cancer: 2 cancer types; Program Number: 1 program; and Predicted Program: microT, miRanda, miRmap, PITA, PicTar and TargetScan (supplementary Table 1).

### Cell culture and treatment

Human umbilical vein endothelial cells (HUVECs) were purchased from the American Type Culture Collection (Manassas, VA, USA). These cells were cultured in endothelial cell growth medium (ECGM; Thermo Fisher, Waltham, MA, USA) supplemented with 10% fetal bovine serum (FBS; Thermo Fisher) in a humidified atmosphere containing 21% O_2,_ 5% CO_2_ at 37°C. For hypoxia treatment, cells were incubated in a hypoxic incubator containing 1% O_2_, 5% CO_2_ and 94% N_2_ at 37°C for 6 or 24 hours [25,26].

### Cell transfection

The plasmid miR-152-3p inhibitor was employed to suppress miR-152-3p expression, and vectors containing short hairpin RNAs against DDX6 (sh-DDX6) were utilized to knockdown DDX6 expression in cells. The above plasmids and vectors as well as their correspondent negative controls (NC inhibitor and sh-NC) were purchased from GenePharma (Shanghai, China). For cell transfection, Lipofectamine 2000 was utilized based on manufacturer’s recommendations [27]. Transfection efficiency was determined utilizing RT-qPCR after 2 days.

### Reverse transcription quantitative polymerase chain reaction (RT-qPCR)

Total RNA in HUVECs was extracted utilizing TRIzol reagent (Invitrogen, Carlsbad, CA, USA) following the manufacturer’s instructions. Reverse Transcription Kits (Promega, Madison, WI, USA) were used for the reverse transcription of RNA into cDNAs. Afterward, a SYBR-Green qPCR Master Mix Kit (Takara, Tokyo, Japan) was applied to perform the quantitative PCR on ABI biosystems (Foster City, CA, USA). RNA levels were calculated by the 2^−∆∆Ct^ method [28]. GAPDH was an internal control for candidate mRNAs while U6 snRNA was a control for miR-152-3p. Sequences of primers are listed in Table 1.

**Table 1.** Sequences of primers used for RT-qPCR

Gene	Sequence (5ʹ→3ʹ)
miR-152-3p forward	TCAGTGCATGACAGAACTTGG
miR-152-3p reverse	CTCTACAGCTATATTGCCAGCCA
DDX6 forward	TTGCTAGCCAAGAAGATTTCTC
DDX6 reverse	ACGATTTCGATGTTCCTGC
ATP2A2 forward	GTATGGCAGGAAAGAAATGCT
ATP2A2 reverse	CTGTCGATACACTTTGCCC
QKI forward	TTTCTGTGGACGCCTAGAG
QKI reverse	TTCCGTACTCTGCTAATTTCTG
SLC25A44 forward	TTCTATGCAGGTTGAGGGC
SLC25A44 reverse	AGATGATTCTGGCCGAGAG
GADD45A forward	AACGACATCAACATCCTGC
GADD45A reverse	AATGTGGATTCGTCACCAG
GAPDH forward	CCTCCTGTTCGACAGTCAG
GAPDH reverse	CATACGACTGCAAAGACCC
U6 forward	CTTTGGCAGCACATATACCA
U6 reverse	CTCATTCAGAGGCCATGCT

### Cell Counting Kit-8 (CCK-8) assay

CCK-8 (Dojindo, Kumamoto, Japan) was employed to measure the viability of HUVECs treated with hypoxia. Approximately 5 × 10^3^ cells were seeded in 96-well plate. After transfection, culture medium was replaced by 100 μL ECGM containing 10 μL CCK-8 reagent for 2 hours of incubation at 37°C. Finally, cells were observed at 570 nm wavelength on a Microplate Reader (Bio-Rad, Hercules, CA, USA) [29].

### Tube formation assay

HUVECs (3 × 10^4^ cells/well) were added with endothelial cell matrix gel (Cell Biolabs, San Diego, CA, USA) according to the manufacturer’s recommendations. Next, cells were added in triplicate to 96-well sterile plates at 37°C for 16 hours. The number of meshes and the length of branches were analyzed using light microscopy [25].

### Western blot analysis

Western blot analysis was conducted according to the previous report [30]. HUVECs were homogenized in RIPA lysis buffer (Beyotime, Shanghai, China) to extract total proteins. The protein concentration was determined by BCA Protein Assay Kits. After the protein contents (50 μg) were separated by 10% SDS-PAGE, they were transferred to PVDF membranes (Millipore, Billerica, MA, USA) for 1 hour of blocking with 5% skim milk at room temperature. Then, the membranes were incubated with primary antibodies (Abcam, Cambridge, UK) at 4°C overnight. Afterward, the membranes were washed and then incubated with secondary antibodies (Abcam) for 1 hour. The protein bands were visualized by enhanced chemiluminescent detection system (Thermo Fisher). GAPDH was set as an internal control. The primary antibodies were against vascular endothelial growth factor A (VEGFA; ab46154; 1:1000), angiopoietin 2 (ANGII; ab155106; 1:5000), ZO-1 (ab276131; 1:1000), occludin (ab242202; 1:1000) and GAPDH (ab9485; 1:2500).

### *Endothelial cell permeability* in vitro *assay*

The assay was conducted according to the previous study [9]. The transwell method was employed to assess endothelial cell permeability. HUVECs were seeded in the upper chamber under hypoxia. The fluorescein isothiocyanate (FITC)-dextran molecules migrating to the lower chamber from the upper chamber through a 3 μm pore size filter were collected. At last, 96-well plate fluorescence reader was employed to measure the fluorescence intensity of these molecules at the wavelength of 495 nm.

### Luciferase reporter assay

The binding site between miR-152-3p and DDX6 was predicted from the starBase. Wild-type (WT) and mutated (MUT) fragments of DDX6 3ʹUTR containing potential binding site with miR-152-3p were synthesized and respectively subcloned into pmirGLO vectors (Promega) to construct pmirGLO-DDX6-WT/MUT reporters. Phusion Site-Directed Mutagenesis Kits (Thermo Fisher) were applied to mutate the predicted binding site. The above reporters were cotransfected with miR-152-3p inhibitor or NC inhibitor into HUVECs using Lipofectamine 3000 reagent (Invitrogen). The luciferase activities of DDX6-WT/MUT were determined utilizing Dual-Glo® Luciferase Assay System Kits (Promega) after 48 hours of cotransfection [31].

### Statistical analysis

Statistical Product and Service Solutions (SPSS) software (Chicago, IL, USA) was utilized for statistical analysis [32]. All assays were performed three times and data were shown as the mean ± standard deviation. Student’s *t* test or one-way analysis of variance followed by Tukey’s *post hoc* test were employed to analyze differences among two or more groups. Statistically significant was defined as *p* < 0.05.

## Results

miR-152-3p was reported to be low-expressed in the hippocampus of ischemia/reperfusion mice according to the previous study. However, the function and mechanism of miR-152-3p have not been explored yet. The study aimed to investigate the effects of miR-152-3p on endothelial cell viability, permeability and angiogenesis. We hypothesized that miR-152-3p might affect endothelial functions of HUVECs by targeting a downstream mRNA. The results indicated that miR-152-3p aggravates endothelial cell permeability and angiogenesis by targeting DDX6 under hypoxia.

### Inhibition of miR-152-3p mitigates hypoxia-induced angiogenesis in HUVECs

**Figure 1.**
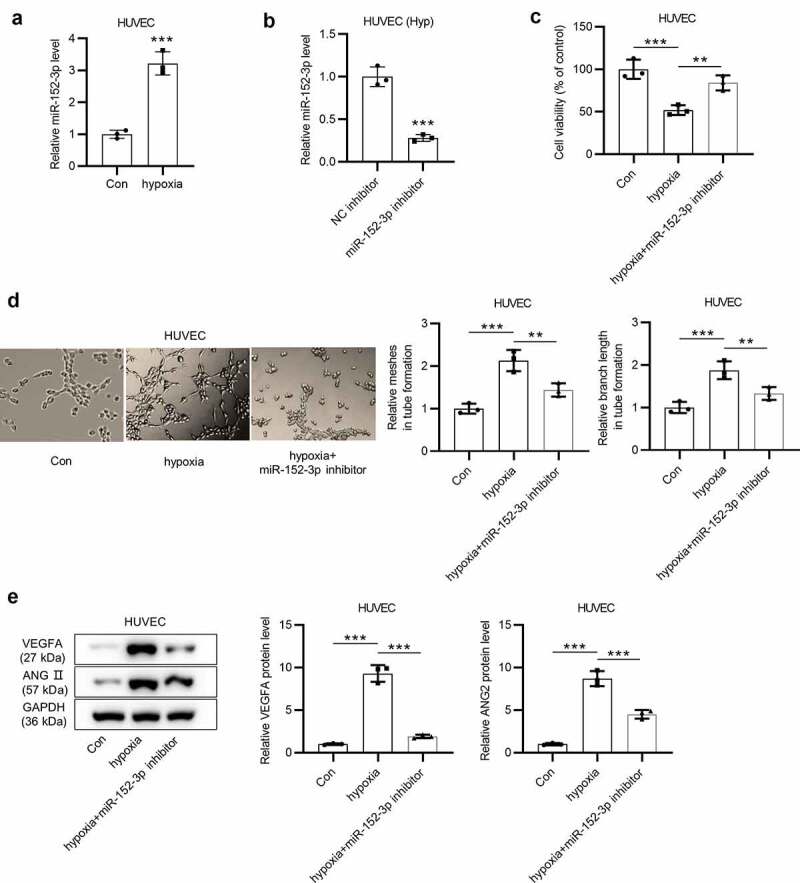
Inhibition of miR-152-3p mitigates hypoxia-induced angiogenesis in HUVECs. (a) miR-152-3p expression in HUVECs under hypoxia was examined by RT-qPCR in thrice. (b) The efficiency of miR-152-3p downregulation was measured by RT-qPCR in triplicate. (c) CCK-8 assays were conducted to detect the viability of HUVECs transfected with miR-152-3p inhibitor under hypoxia. each experiment was repeated three times. (d) Tube formation assays were applied to determine effects of mir-152-3p inhibitor on angiogenesis. the experiment was conducted in triplicate. (e) Western blot analyses were conducted three times to examine levels of angiogenesis-associated proteins (VEGFA and ANGII). **p < 0.01, ***p < 0.001

The expression of miR-152-3p in HUVECs under normoxia and hypoxia, and its effects on cell viability, tube formation and endothelial cell permeability were investigated. RT-qPCR exhibited that miR-152-3p expression was significantly (***p < 0.001) increased in HUVECs under hypoxia (Figure 1a). Next, we suppressed miR-152-3p expression by transfection of miR-152-3p inhibitor into HUVECs under hypoxia to conduct loss-of-function assays. As shown by RT-qPCR, miR-152-3p expression was successfully (***p < 0.001) decreased in cells treated with hypoxia (Figure 1b). CCK-8 assays illustrated that miR-152-3p inhibition partially (**p < 0.01) reversed the decrease (***p < 0.001) in cell viability mediated by hypoxia (Figure 1c). Tube formation assays were performed to evaluate angiogenesis *in vitro*. As a result, miR-152-3p inhibition significantly (**p < 0.01) reversed hypoxia-induced increase (***p < 0.001) in tube formation ability of HUVECs (Figure 1d). According to western blot analysis, miR-152-3p inhibition markedly (***p < 0.001) reversed hypoxia-mediated upregulation (***p < 0.001) of vascular endothelial growth factor A (VEGFA) and angiopoietin-2 (ANGII) protein levels in cells (Figure 1e). Overall, miR-1523-p was high-expressed in HUVECs under hypoxia. Inhibition of miR-152-3p reverses hypoxia-induced decrease in cell viability and hypoxia-activated tube formation of HUVECs.

### Inhibition of miR-152-3p alleviates hypoxia-induced endothelial permeability of HUVECs

**Figure 2.**
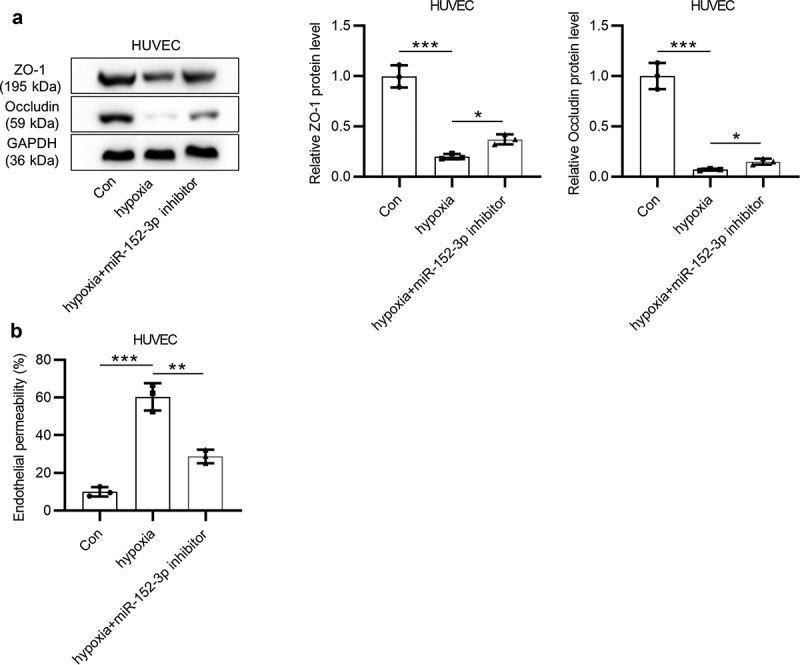
Inhibition of miR-152-3p alleviates hypoxia-induced endothelial permeability of HUVECs. (a) Western blot analysis was employed to examine levels of tight junction proteins (ZO-1 and occludin) in HUVECs under hypoxia. Each experiment was performed in triplicate. (b) Endothelial permeability of indicated cells was detected utilizing endothelial cell permeability *in vitro* assays. the assay was performed in thrice. *p < 0.05, **p < 0.01, ***p < 0.001

Western blot analysis and endothelial cell permeability *in vitro* assays were conducted to measure endothelial cell permeability. As shown by western blot analysis, miR-152-3p inhibitor partially (*p < 0.05) reversed hypoxia-mediated decrease (***p < 0.001) in ZO-1 and occludin protein levels in cells (Figure 2a). Endothelial cell permeability *in vitro* assays revealed that miR-152-3p inhibitor partially (**p < 0.01) reversed the promotion (***p < 0.001) of endothelial permeability induced by hypoxia (Figure 2b). The results suggested that inhibition of miR-152-3p alleviates endothelial permeability promoted by hypoxia.

### DDX6 is targeted by miR-152-3p

**Figure 3.**
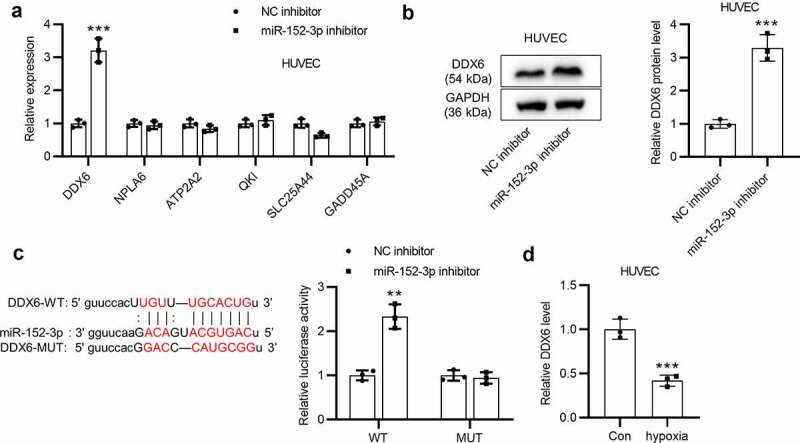
DDX6 is targeted by miR-152-3p. (a) Expression levels of candidate mRNAs in HUVECs transfected with miR-152-3p inhibitor or NC inhibitor were examined utilizing RT-qPCR in thrice. (b) Western blot analyses were conducted three times to measure effects of miR-152-3p inhibitor on the protein level of DDX6 in cells. (c) The possible binding site between miR-152-3p and DDX6 was predicted from the starBase, and luciferase reporter assays were performed in thrice to explore the interaction between miR-152-3p and DDX6. (d) DDX6 expression in HUVECs under hypoxia was examined utilizing RT-qPCR analysis in triplicate. **p < 0.01, ***p < 0.001

Subsequently, we hypothesized that miR-152-3p might contribute to endothelial cell dysfunction due to the miRNA-target mechanism. To verify the hypothesis, bioinformatics analysis was performed to seek for possible mRNAs possessing binding capacity for miR-152-3p. Six mRNAs (DDX6, NPLA6, ATP2A2, QKI, SLC25A44 and GADD45A) binding to miR-152-3p were predicted utilizing the starBase (supplementary Table 1). RT-qPCR was employed to assess expression levels of these candidate genes in HUVECs with transfection of miR-152-3p inhibitor or NC inhibitor. Compared with the expression of other genes, DDX6 expression was significantly (***p < 0.001) upregulated by miR-152-3p inhibitor in HUVEC (Figure 3a). Next, we probed the effects of silencing miR-152-3p on DDX6 protein level in cells. As suggested by western blot analysis, miR-152-3p inhibitor markedly (***p < 0.001) upregulated the protein level of DDX6 in HUVECs (Figure 3b). Afterward, we explored the interaction between miR-152-3p and DDX6. The potential binding site between miR-152-3p and DDX6 was predicted with the starBase, and the sequence of DDX6 was mutated. Luciferase reporter assays revealed that the luciferase activity of DDX6-WT was markedly (**p < 0.01) increased by miR-152-3p inhibitor while no significant changes were examined in that of DDX6-MUT (Figure 3c). Additionally, RT-qPCR analysis suggested that DDX6 expression was significantly (***p < 0.001) downregulated in HUVECs treated with hypoxia (Figure 3d). In conclusion, miR-152-3p targets DDX6 and negatively modulates DDX6 expression in HUVECs.

### miR-152-3p promotes tube formation of HUVECs by targeting DDX6

**Figure 4.**
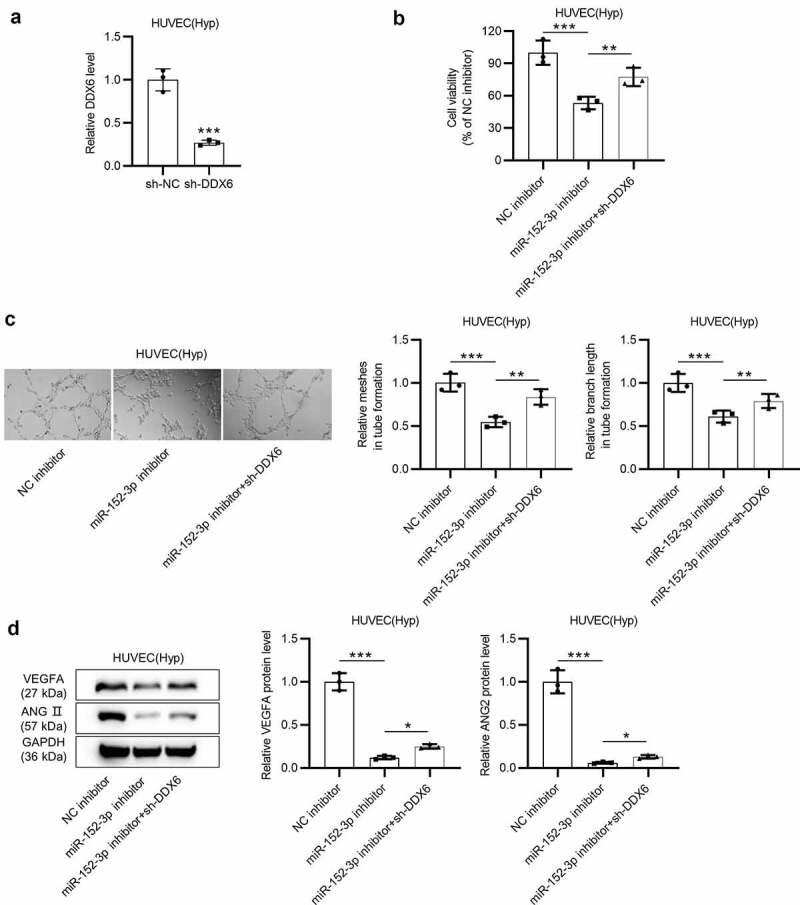
miR-152-3p promotes tube formation of HUVECs by targeting DDX6. (a) The knockdown efficiency of DDX6 in HUVECs under hypoxia was detected by RT-qPCR in thrice. (b) CCK-8 assays were carried out three times to probe effects of miR-152-3p inhibition and DDX6 silencing on cell viability. (c) The angiogenesis in cells with different transfection was measured by tube formation assays. Each experiment was repeated in triplicate. (d) Western blot analyses were employed to probe protein levels of angiogenesis-associated factors in cells with transfection of miR-152-3p inhibitor and sh-DDX6. Each analysis was performed three times. *p < 0.05, **p < 0.01, ***p < 0.001

To validate whether miR-152-3p suppresses cell viability while accelerates tube formation under hypoxia by targeting DDX6, rescues assays were carried out in HUVECs transfected with miR-152-3p inhibitor, NC inhibitor or co-transfected with sh-DDX6 and miR-152-3p inhibitor under hypoxia. First, the knockdown efficiency of DDX6 expression was detected utilizing RT-qPCR analysis, which suggested a significant (***p < 0.001) decrease in DDX6 expression in cells (Figure 4a). CCK-8 assays revealed that DDX6 knockdown partially (**p < 0.01) rescued the decrease (***p < 0.001) in cell viability induced by miR-152-3p inhibition (Figure 4b). As shown in tube formation assays, DDX6 silencing partially (**p < 0.01) rescued the suppressive effect (***p < 0.001) of miR-152-3p inhibitor on tube formation in cells (Figure 4c). The protein levels of VEGFA and ANGII were greatly (***p < 0.001) downregulated by miR-152-3p inhibition and then partially (*p < 0.05) reversed by DDX6 knockdown in cells (Figure 4d). The above results verified that miR-152-3p inhibits cell viability while promotes angiogenesis under hypoxia by targeting DDX6.

### miR-152-3p facilitates endothelial permeability of HUVECs by downregulating DDX6 expression

**Figure 5.**
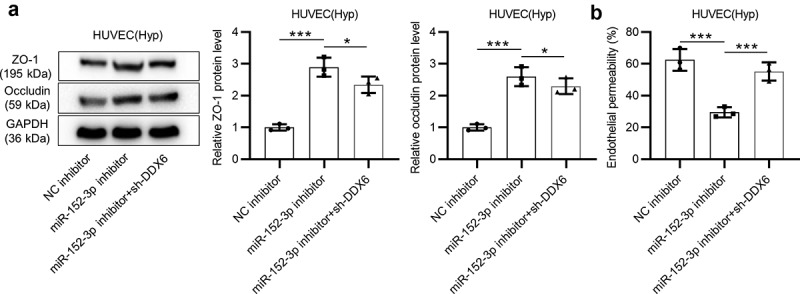
miR-152-3p facilitates endothelial permeability of HUVECs by downregulating DDX6 expression. (a) Protein levels of ZO-1 and occludin in cells with transfection of NC inhibitor, miR-152-3p inhibitor or miR-152-3p inhibitor + sh-DDX6 were examined by western blot analyses. each analysis was performed in thrice. (b) The rescue effect of DDX6 on the decrease in cell permeability induced by miR-152-3p inhibition was probed utilizing endothelial cell permeability *in vitro* assays. Each experiment was performed in thrice. *p < 0.05, ***p < 0.001

Western blot analyses and endothelial cell permeability *in vitro* assays were performed to verify whether miR-152-3p accelerates endothelial cell permeability by targeting DDX6. Protein levels of tight junction markers (ZO-1 and occludin) in HUVECs under hypoxia were examined by western blot. DDX6 depletion partially (*p < 0.05) reversed the upregulation (***p < 0.001) of ZO-1 and occludin protein levels mediated by miR-152-3p inhibition in cells (Figure 5a). According to endothelial cell permeability *in vitro* assays, DDX6 knockdown partially (***p < 0.001) rescued the inhibitory effect (***p < 0.001) of miR-152-3p inhibition on endothelial cell permeability (Figure 5b). Overall, miR-152-3p facilitates endothelial permeability of HUVECs by downregulating DDX6 expression.

## Discussion

Stroke is a deadly disease that is threatening lives worldwide [33]. Endothelial permeability, inflammation and oxidative stress are factors influencing the outcome of stroke, and angiogenesis is critical to recover blood supply after stroke [34,35]. MicroRNAs (miRNAs) are deemed as diagnostic and therapeutic biomarkers for stroke [36], and its regulation of endothelial cell functions was extensively reported [37–39]. Previously, miR-152-3p was found to be low-expressed in the hippocampus of ischemia/reperfusion and postconditioning mice [22]. In addition, decreased miR-152-3p was detected in patients who suffered acute ischemic stroke with National Institute of Health stroke scale (NIHSS) score≥7, suggesting that decreased miR-152-3p is related to the severity of endothelial injury [23]. Moreover, miR-152-3p is associated with detrimental microvascular functions in oxygen-induced retinopathy [40].

Herein, we discovered that miR-152-3p exhibited high expression in HUVECs under hypoxia. Loss-of-function assays revealed that miR-152-3p inhibition reversed hypoxia-mediated increase in endothelial cell permeability and angiogenesis.

Angiogenesis is a compensatory response to the reduction of oxygen, which contributes to the restoration of blood flow and thereby promotes the recovery of ischemic stroke [7]. Vascular endothelial growth factor A (VEGFA) and angiopoietin 2 (ANGII) are essential factors related to angiogenesis [41–43]. In our study, VEGFA and ANGII protein levels in cells and tube formation ability of HUVECs were significantly upregulated by hypoxia while miR-152-3p inhibition reversed the increase, suggesting that miR-152-3p promotes angiogenesis under hypoxia.

Neurological functions can be damaged by the increase in endothelial permeability [35]. Tight junctions existing between cerebral endothelial cells form a diffusion barrier that prevents most blood-borne substances from entering the brain [44]. The barrier is known as blood-brain-barrier (BBB). As a selectively permeable cellular monolayer, the BBB affects the homeostasis within the brain [34,45]. Tight junction proteins such as occludin and ZO-1 are strongly related with BBB stability [46]. Herein, we found that miR-152-3p inhibition rescued hypoxia-induced decrease in ZO-1 and occludin expression in HUVECs. Moreover, endothelial cell permeability *in vitro* assays suggested that inhibiting miR-152-3p expression reversed the promotion of endothelial permeability mediated by hypoxia. The results suggest that miR-152-3p promotes endothelial cell permeability under hypoxia.

Mechanistically, miRNAs regulate mRNA expression post-transcriptionally by binding to 3ʹUTR of mRNAs [47]. In previous studies, miR-152-3p was discovered to modulate cell proliferation and invasion by targeting forkhead box F1 (FOXF1) in keloid fibroblasts [48]. The miR-152-3p/Gadd45 correlation is also investigated in PC12-Derived nerve cells [49]. To explore the downstream molecule of miR-152-3p, mRNAs binding with miR-152-3p were predicted using the starBase, and DDX6 was finally selected for further study due to its increased expression mediated by miR-152-3p inhibition in cells. DDX6, a member of the DDX RNA helicase family, is an RNA-binding protein that correlates with different aspects of gene expression regulation [50]. DDX6 was demonstrated to increase the activity of miRNA let-7a in neural stem cells [51]. DDX6 was found to increase the activity of miR-21 and miR-124 by interacting with Tau protein in tauopathies [52]. Functions of DDX6 in vascular endothelial cells still require more investigation. Herein, DDX6 expression was downregulated in HUVECs under hypoxia. MiR-152-3p inhibition upregulated mRNA expression and protein levels of DDX6 in cells. According to luciferase reporter assays, miR-152-3p directly targeted the 3ʹUTR of DDX6 in cells. In addition, rescue assays showed that DDX6 knockdown reversed the alleviation of angiogenesis and endothelial permeability mediated by miR-152-3p inhibition. The results indicated that miR-152-3p promotes angiogenesis and endothelial permeability by downregulating DDX6 expression.

Furthermore, DDX6 was reported to have a strong binding capacity for vascular endothelial growth factor (VEGF) 5ʹ-UTR [25]. VEGF contributes to the formation of blood vessels [53]. Under hypoxic conditions, VEGF expression is mediated by the increase in mRNA synthesis, stability and translation. Elevated VEGF mRNA synthesis under hypoxia is induced by hypoxia-inducible factor 1 (HIF-1) which is a transcription factor composing of a constitutive β-subunit and an O_2_-labileα-subunit (HIF-1α) [25]. HIF-1α is essential for the maintenance of oxygen homeostasis [54]. Another study reveals that HIF-1α mRNA is released from the P-bodies or does not enter into P-bodies through decreased DDX6 [54]. Herein, we did not further explore how DDX6 affects angiogenesis, endothelial cell permeability and viability under hypoxia. We hypothesized that the relationship between DDX6 and HIF-1α might be the answer, which will be further explored in future studies.

In conclusion, miR-152-3p aggravates angiogenesis in vascular endothelial cells and promotes endothelial cell permeability by targeting DDX6 under hypoxia. The study may provide novel insight into miRNA investigation in ischemic stroke. However, how hypoxia treatment affects miR-152-3p and DDX6 expression in HUVECs are not investigated and *in vivo* animal experiments have not been designed in the study, which will be included in our future studies. In addition, the upstream gene that may regulate miR-152-3p expression and the possible signaling pathway mediated by DDX6 in HUVECs will be investigated in our future studies.

## Conclusion

miR-152-3p is highly expressed in HUVECs under hypoxia. miR-152-3p inhibition elevates the decrease in cell viability induced by hypoxia. miR-152-3p inhibition mitigates the activation of angiogenesis and endothelial cell permeability under hypoxia. Mechanistically, DDX6 is targeted by miR-152-3p in HUVECs. DDX6 deficiency reverses the effects of miR-152-3p inhibition on angiogenesis, endothelial cell viability and permeability under hypoxia. Overall, miR-152-3p aggravates vascular endothelial cell dysfunction by targeting DDX6 under hypoxia.

## Supplementary Material

Supplemental Material

## HIGHLIGHTS

1. miR-152-3p is high-expressed in HUVECs under hypoxia.
2. Inhibition of miR-152-3p mitigates hypoxia-activated angiogenesis.
3. Inhibition of miR-152-3p alleviates hypoxia-induced endothelial cell permeability.
4. DDX6 is targeted by miR-152-3p.
5. DDX6 depletion reverses effects of miR-152-3p inhibition on cell dysfunction.
